# Skipping Breakfast and Subsequent Overweight/Obesity in Children: A Nationwide Prospective Study of 2.5- to 13-year-old Children in Japan

**DOI:** 10.2188/jea.JE20200266

**Published:** 2021-07-05

**Authors:** Yuri Yaguchi-Tanaka, Takahiro Tabuchi

**Affiliations:** 1 Department of Education, Art, and Science, Yamagata University, Yamagata, Japan; 2 Cancer Control Center, Osaka International Cancer Institute, Osaka, Japan

**Keywords:** skipping breakfast, longitudinal study, children, overweight/obesity

## Abstract

**Background:**

Few longitudinal studies have examined the association between skipping breakfast and overweight/obesity in pre-elementary school children. Furthermore, this association may differ between boys and girls. The main objective of this study was to assess whether skipping breakfast in early childhood was associated with later incidence of overweight/obesity, with stratification by gender, using data on children aged 2.5 to 13 years old in The Longitudinal Survey of Newborns in the 21^st^ century.

**Methods:**

We examined the associations between skipping breakfast at 2.5 years old and overweight/obesity at 2.5 (*n* = 34,649), 4.5 (*n* = 35,472), 7 (*n* = 31,266), 10 (*n* = 31,211), and 13 (*n* = 28,772) years old. To estimate adjusted odds ratios (ORs) with 95% confidence intervals (CIs) of overweight/obesity by each age (2.5, 4.5, 7, 10, and 13 years), a multivariate logistic regression was used adjusting for time-invariant and time-varying covariates.

**Results:**

At the age of 2.5 years, 11.0% of boys and 12.2% of girls were skipping breakfast. In fully adjusted models, skipping breakfast at 2.5 years old was not significantly associated with overweight/obesity at 2.5 and 4.5 years old, but was significantly associated with overweight/obesity at 7 and 10 years old, in both sexes. Skipping breakfast at 2.5 years old was significantly associated with overweight/obesity at 13 years old in boys (OR 1.38; 95% CI, 1.17–1.62), but not in girls (OR 1.21; 95% CI, 0.98–1.49).

**Conclusions:**

Skipping breakfast in early childhood increased overweight/obesity in later childhood, but there may be gender differences in the association.

## INTRODUCTION

Skipping breakfast has been reported as an unhealthy dietary habit associated with a poorer quality diet among children and adolescents.^1^^,^^2^ A positive relationship between skipping breakfast and overweight and/or obesity in children and adolescents has been observed in cross-sectional studies^3^^–^^6^ and a meta-analysis.^7^ Most longitudinal studies indicated that skipping breakfast over time was associated with weight gain in school children and adolescents.^8^^–^^13^ Overweight and obesity in childhood or adolescence significantly increased the risk of premature mortality and adult morbidity, particularly cardiometabolic diseases.^14^^,^^15^ Therefore, habitual eating breakfast from childhood is important for preventing obese or metabolic disease in adults. However, longitudinal studies that investigated the association between skipping breakfast and overweight/obesity of kindergarten and nursery school age children are scarce,^16^^–^^19^ and results of these studies were not consistent. Early childhood breakfast skipping was associated with subsequent obesity in two studies,^16^^,^^17^ but a further study showed no association.^18^ Skipping breakfast at 4 years old was only associated with a higher body fat mass at 6 years old in one other study.^19^

In Japan, The Longitudinal Survey of Newborns in the 21^st^ Century (hereafter ‘the Newborns Survey’), a nationally representative longitudinal study, also reported children’s breakfast eating status: 12–19% of children aged 2.5 to 4.5 years old skipped breakfast.^20^^,^^21^ Using this survey data, a previous study indicated that skipping breakfast at 2.5 years old was associated with childhood overweight/obesity up to elementary school age.^20^ However, some research gaps remain in the analysis: neither stratification by gender nor the outcomes at junior high school age were included. As for gender difference, the prevalence of overweight/obesity tends to increase with age in boys, but this tendency was not observed in girls.^22^^,^^23^ Furthermore, this gender difference increased in children of junior high school age and older.^22^^,^^23^ Therefore, the association between skipping breakfast and subsequent overweight/obesity may differ by gender, especially after junior high school age. However, other studies did not show identical results in terms of prevalence with age in both sexes.^24^^,^^25^ Thus, the objective of this study was to assess whether skipping breakfast in early childhood was associated with prevalence of overweight and obesity in later childhood, with stratification by gender, using data from children aged 2.5 years old to junior high school age in the Newborns Survey.

## METHODS

### Study population

The data used for this study were taken from the Newborns Survey, which was conducted by the Japanese Ministry of Health, Labour and Welfare from 2001 to 2013. The study sample included all infants born in Japan during the periods January 10–17, 2001, and July 10–17, 2001, using national birth records (*n* = 53,575). Questionnaires were mailed when the infants were 0.5 years old and 47,015 people responded (response rate, 87.8%). Follow-up surveys were conducted at the ages of 1.5, 2.5, 3.5, 4.5, 5.5, 7, 8, 9, 10, 11, 12, and 13 years old. Anyone who did not respond to the survey for 2 consecutive years was excluded from the survey the following year. Respondent numbers for each survey were: 43,925 (82.0%), 42,812 (79.9%), 41,559 (77.6%), 39,817 (74.3%), 38,537 (71.9%), 36,785 (68.7%), 36,136 (67.4%), 35,264 (65.8%), 34,124 (63.7%), 32,913 (61.4%), 32,065 (59.9%), and 30,331 (56.6%), respectively. During the follow-up period, which lasted up to the age of 13 years, the response rate from each previous year ranged from 95–98%; the cumulative response rate from the 3rd to 13th survey was 70.8% (13 years old respondent numbers 30,331, 2.5 years old respondent numbers 42,812).^21^

All respondents provided consent for the Newborns Survey defined by the Japanese government, and we obtained permission from the Ministry of Health, Labor and Welfare to use the survey data for this study. Information on children’s birthweight and maternal and paternal age was obtained from birth records. Data on paternal and maternal educational status were collected at the age of 1.5 years old (ie, second survey). When the children were 0.5 years old, their parents were asked about breastfeeding status. As a behavioral factor, we used number of hours watching television and playing computer games per weekday. Information on living with a grandparent was obtained for every age. The study was approved by the ethical committee of the Osaka International Cancer Institute (No. 1508119060).

### Anthropometry

In each questionnaire, parents were required to provide anthropometric measurements, including weight (to the nearest 0.1 kg) and height (to the nearest 0.1 cm) at that time. BMI was calculated using the following formula: weight (kg)/height (m)^2^. Childhood overweight and obesity were defined using the International Obesity Task Force BMI cut points, which were derived from six large, nationally representative cross-sectional surveys on growth. These cut-off points were defined for children (2–18 years of age), based on a sex-specific BMI corresponding to the 25 kg/m^2^ cut-off points at the age of 18 years, based on the BMI centile curves.^26^

### Breakfast eating habits

Parents were asked about skipping breakfast in the 2^nd^ and 9^th^ surveys (corresponding to children’s ages 1.5 and 9 years old) as follows^20^: “Do you usually eat breakfast? Yes or No.” Information concerning children skipping breakfast was collected in the 3^rd^ through 13^th^ surveys (corresponding to children’s age from 2.5 to 13 years old), with the exception of the 6^th^ survey (corresponding to children’s age of 5.5 years old). Although questions regarding skipping breakfast varied between surveys, skipping breakfast was classified as a binary variable (yes or no): “Has your child ever skipped breakfast? Yes or No” in the 3^rd^ and 4^th^ surveys; “Has your child skipped breakfast? Yes (usually or sometimes) or No (never)” in the 5^th^ survey; “Does your child usually skip breakfast? Yes (usually or sometimes) or No (rarely or not at all)” in the 7^th^ and 8^th^ surveys; “Does your child usually eat breakfast? Yes (no breakfast skipping) or No (breakfast skipping)” in the 9^th^ and 10^th^ surveys; “Do you usually eat breakfast? Yes (no breakfast skipping) or No (breakfast skipping)” in the 11^th^ and 12^th^ surveys, “Do you usually eat breakfast? Yes (no breakfast skipping) or No (breakfast skipping sometimes or usually)” in the 13^th^ survey. Parents were asked these questions in the 3^rd^ through the 10^th^ surveys, while the children were asked these questions in the 11^th^ through the 13^th^ survey.

### Statistical analysis

Using SPSS ver. 24.0 (IBM Japan Inc., Tokyo, Japan), we conducted all analyses separately by sex. We examined the associations between skipping breakfast at 2.5 years old and overweight/obesity at 2.5, 4.5, 7, 10, and 13 years old. In the Japanese education system, all children must enter elementary school at 6 years of age, and junior high school at 12 years; 94.6% of 4-year-old children attend pre-school (kindergarten and nursery school).^27^ We chose these ages bearing in mind the possibility of changes in school environment (ie, kindergarten and nursery school, first half of elementary school, second half of elementary school, and junior high school).

We tested the differences between skipping breakfast (yes/no) at 2.5 years old and the prevalence of overweight/obesity and skipping breakfast (yes/no) children after 4.5 years old by χ^2^ tests and residual analysis. To estimate adjusted odds ratios (ORs) with 95% confidence intervals (CIs) of overweight/obesity by each age (2.5, 4.5, 7, 10, and 13 years old), a multivariable logistic regression was used with stratification by gender, adjusting for time-invariant and time-varying covariates. It has been shown that the risk factors for childhood overweight/obesity were birth weight, breastfeeding, parental age, parental education, as time-invariant sociodemographic characteristics.^20^^,^^28^^,^^29^ Furthermore, it has been shown that the risk factors for childhood overweight/obesity were skipping breakfast, living with grandparents, time spent watching television and time spent playing computer games, as time-varying covariates on sociodemographic characteristics.^3^^,^^28^ Therefore, the time-invariant confounders we used maternal age at birth, paternal age at birth, birthweight, maternal educational level, paternal educational level, and breastfeeding exclusiveness.^20^^,^^28^^,^^29^ Time-varying confounders included skipping breakfast (yes/no), living with a grandparent (yes/no), hours spent watching television, and hours spent playing computer games at each analytical age in the analysis.^3^^,^^28^ Since data for hours spent watching television at age 13 years old, and hours spent playing computer games at age 2.5 and 13 years old were not surveyed, we excluded these data from the analysis. In addition to gender-stratified analyses, we also analyzed pooled data of boys and girls to investigate gender differences. To account for the interaction effect between sex and skipping/eating breakfast in an additional analysis, one interaction term was used in each model, given by the product of the two variables (ie, boys/girls, skipping breakfast/eating breakfast). The missing values were categorized as a dummy variable and were added to the analysis. We examined the multicollinearity of variables before the multivariable logistic regression analysis. Multicollinearity in multivariate models was assessed using the definition of having condition indices ≥30 and at least two variables with variance decomposition proportions >0.5. Probability values for statistical tests were two-tailed, and *P* < 0.05 or |Z| > 1.96 was regarded as statistically significant.

**Figure 1.**
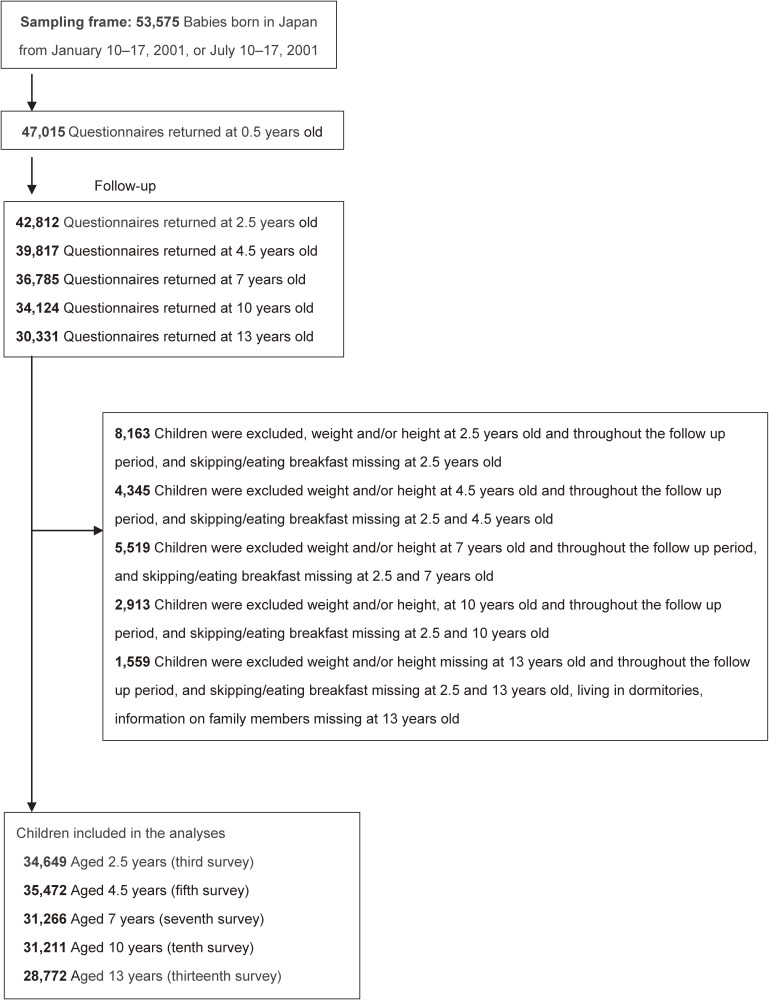
Flow diagram for selection of participant children from 2001 to 2013

Of those who responded to the second survey (*n* = 42,812), after exclusion of children with missing values for weight or height and skipping/eating breakfast at each analytic age and throughout the follow up period, and missing for skipping breakfast at 2.5 years old and at each analytic age; 34,649, 35,472, 31,266 and 31,211 remaining samples were analyzed at 2.5, 4.5, 7, and 10 years old, respectively (Figure 1). Children who lived in a school dormitory or had missing information on family members were excluded from the analysis; 28,772 children were analyzed at 13 years old (Figure 1).

## RESULTS

Time-invariant characteristics of study participants at 2.5 years old are shown in Table 1. Table 2 shows the time-varying factors at 2.5, 4.5, 7, 10 and 13 years old. At age 2.5 years old, 11.0% of boys and 12.2% of girls were skipping breakfast. Among boys, prevalence of overweight/obesity gradually increased from 7.9% at 2.5 years to over 10% at 10–13 years old. On the other hand, among girls, the prevalence of overweight/obesity decreased from 10.0% at 2.5 years to 5.8% at 13 years.

**Table 1.** Time-invariant characteristics of study subjects at 2.5 years old

		Boys	Girls	Total
		*n* = 18,013	*n* = 16,636	*n* = 34,649
Birthweight, g^a^	<2,500	1,370 (7.6%)	1,583 (9.5%)	2,953 (8.5%)
	2,500–2,999	5,968 (33.1%)	6,738 (40.5%)	12,706 (36.7%)
	3,000–3,499	8,039 (44.6%)	6,708 (40.3%)	14,747 (42.6%)
	≥3,500	2,630 (14.6%)	1,604 (9.6%)	4,234 (12.2%)
	Missing	6 (0.0%)	3 (0.0%)	9 (0.0%)

Breastfeeding exclusiveness^b^	Exclusive breastfeeding	3,810 (21.2%)	3,668 (22.0%)	7,478 (21.6%)
	Mixed feeding	13,142 (73.0%)	12,045 (72.4%)	25,187 (72.7%)
	Exclusive formula feeding	1,061 (5.9%)	923 (5.5%)	1,984 (5.7%)

Maternal age at birth^a^	≤24	1,543 (8.6%)	1,426 (8.6%)	2,969 (8.6%)
	25–29	6,140 (34.1%)	5,671 (34.1%)	11,811 (34.1%)
	30–34	7,338 (40.7%)	6,746 (40.6%)	14,084 (40.6%)
	≥35	2,992 (16.6%)	2,793 (16.8%)	5,785 (16.7%)

Paternal age at birth^a^	≤24	946 (5.3%)	857 (5.2%)	1,803 (5.2%)
	25–29	4,363 (24.2%)	4,116 (24.7%)	8,479 (24.5%)
	30–34	6,723 (37.3%)	6,201 (37.3%)	12,924 (37.3%)
	≥35	5,819 (32.3%)	5,279 (31.7%)	11,098 (32.0%)
	Missing	162 (0.9%)	183 (1.1%)	345 (1.0%)

Maternal educational level^c^	Junior high school	580 (3.2%)	558 (3.4%)	1,138 (3.3%)
	High school	6,837 (38.0%)	6,382 (38.4%)	13,219 (38.2%)
	Junior or career college	7,557 (42.0%)	6,741 (40.5%)	14,298 (41.3%)
	University or higher education	2,542 (14.1%)	2,506 (15.1%)	5,048 (14.6%)
	Other/missing	497 (2.8%)	449 (2.7%)	946 (2.7%)

Paternal educational level^c^	Junior high school	1,042 (5.8%)	944 (5.7%)	1,986 (5.7%)
	High school	6,954 (38.6%)	6,386 (38.4%)	13,340 (38.5%)
	Junior or career college	2,753 (15.3%)	2,553 (15.3%)	5,306 (15.3%)
	University or higher education	6,608 (36.7%)	6,134 (36.9%)	12,742 (36.8%)
	Other/missing	656 (3.6%)	619 (3.7%)	1,275 (3.7%)

^a^This information collected from birth records.

^b^This information collected from survey at 0.5 years old.

^c^This information collected from survey at 1.5 years old.

**Table 2.** Time-varying factors of study subjects

			2.5 years old	4.5 years old	7 years old	10 years old	13 years old
Boys	Number		18,013	18,463	16,223	16,119	14,911

	Overweight/Obese	No	16,581 (92.1%)	17,302 (93.7%)	14,723 (90.8%)	14,053 (87.2%)	13,410 (89.9%)
		Yes	1,432 (7.9%)	1,161 (6.3%)	1,500 (9.2%)	2,066 (12.8%)	1,501 (10.1%)

	Skipping breakfast	No	16,029 (89.0%)	14,944 (80.9%)	15,672 (96.6%)	15,981 (99.1%)	13,854 (92.9%)
		Yes	1,984 (11.0%)	3,519 (19.1%)	551 (3.4%)	138 (0.9%)	1,057 (7.1%)

	Living with a grandparent	No	13,952 (77.5%)	14,203 (76.9%)	12,319 (75.9%)	12,402 (76.9%)	11,688 (78.4%)
		Yes	4,061 (22.5%)	4,260 (23.1%)	3,904 (24.1%)	3,717 (23.1%)	3,223 (21.6%)

	Watching television (hours per day)	<1	1,859 (10.3%)	2,005 (10.9%)	4,450 (27.4%)	3,134 (19.4%)	—
		1–<2	6,638 (36.9%)	5,446 (29.5%)	7,412 (45.7%)	6,998 (43.4%)	—
		2–<3	2,536 (14.1%)	6,568 (35.6%)	3,346 (20.6%)	4,197 (26.0%)	—
		≥3	6,600 (36.6%)	4,255 (23.0%)	987 (6.1%)	1,731 (10.7%)	—
		Missing	380 (2.1%)	189 (1.0%)	28 (0.2%)	59 (0.4%)	—

	Playing computer games (hours per day)	<1	—	16,578 (89.8%)	12,749 (78.6%)	10,986 (68.0%)	—
		1–<2	—	1,464 (7.9%)	2,902 (17.9%)	4,176 (25.9%)	—
		≥2	—	379 (2.1%)	480 (3.0%)	863 (5.4%)	—
		Missing	—	42 (0.2%)	92 (0.6%)	112 (0.7%)	—

Girls	Number		16,636	17,009	15,043	15,092	13,861

	Overweight/Obese	No	14,969 (90.0%)	15,458 (90.9%)	13,657 (90.8%)	13,778 (91.3%)	13,063 (94.2%)
		Yes	1,667 (10.0%)	1,551 (9.1%)	1,386 (9.2%)	1,314 (8.7%)	798 (5.8%)

	Skipping breakfast	No	14,599 (87.8%)	13,697 (80.5%)	14,547 (96.7%)	14,967 (99.2%)	12,872 (92.9%)
		Yes	2,037 (12.2%)	3,312 (19.5%)	496 (3.3%)	125 (0.8%)	989 (7.1%)

	Living with a grandparent	No	12,907 (77.6%)	13,081 (76.9%)	11,498 (76.4%)	11,714 (77.6%)	10,912 (78.7%)
		Yes	3,729 (22.4%)	3,928 (23.1%)	3,545 (23.6%)	3,378 (22.4%)	2,949 (21.3%)

	Watching television (hours per day)	<1	1,692 (10.2%)	2,139 (12.6%)	4,450 (29.6%)	3,053 (20.2%)	—
		1–<2	6,140 (36.9%)	5,162 (30.3%)	6,621 (44.0%)	6,254 (41.4%)	—
		2–<3	2,340 (14.1%)	5,815 (34.2%)	3,025 (20.1%)	4,000 (26.5%)	—
		≥3	6,147 (36.9%)	3,721 (21.9%)	923 (6.1%)	1,742 (11.5%)	—
		Missing	317 (1.9%)	172 (1.0%)	24 (0.2%)	43 (0.3%)	—

	Playing computer games (hours per day)	<1	—	16,346 (96.1%)	13,816 (91.8%)	13,255 (87.8%)	—
		1–<2	—	528 (3.1%)	1,022 (6.8%)	1,578 (10.5%)	—
		≥2	—	112 (0.7%)	93 (0.6%)	179 (1.2%)	—
		Missing	—	23 (0.1%)	112 (0.7%)	80 (0.5%)	—

Prevalence of skipping breakfast (yes/no) and overweight/obesity after the age of 4.5 years old, according to skipping breakfast status at 2.5 years old, is shown in Table 3. Compared with who did not skip breakfast at 2.5 years old, those who did skip breakfast at 2.5 years old were likely to also skip breakfast at 4.5, 7, 10 and 13 years old. In 4.5, 7, 13 years old boys and girls over 7 years, the lowest prevalence of overweight/obesity was among children who ate both breakfast at 2.5 years old and at each age.

**Table 3.** Prevalence of skipping breakfast and overweight/obesity after 4.5 years old, according to skipping breakfast status at 2.5 years old

		2.5 years old skipping breakfast		skipping breakfast at each age^a^		*P*-value^b^	overweight/obese at each age^c^	*P*-value^b^
Boys								
	4.5 years old	No	16,426	No	13,898 (84.6%)^d^	<0.001	818 (5.9%)	<0.001
				Yes	2,528 (15.3%)^e^		204 (8.1%)	
		Yes	2,037	No	1,046 (51.3%)^d^		62 (5.9%)	
				Yes	991 (48.6%)^e^		77 (7.8%)	

	7 years old	No	14,506	No	14,107 (97.2%)^d^	<0.001	1,261 (8.9%)	0.006
				Yes	399 (2.8%)^e^		46 (11.5%)	
		Yes	1,717	No	1,565 (91.1%)^d^		177 (11.3%)	
				Yes	152 (8.9%)^e^		16 (10.5%)	

	10 years old	No	14,424	No	14,322 (99.3%)^d^	<0.001	1,781 (12.3%)	<0.001
				Yes	102 (0.7%)^e^		12 (11.8%)	
		Yes	1,695	No	1,657 (97.9%)^d^		262 (15.8%)	
				Yes	36 (2.1%)^e^		11 (30.6%)	

	13 years old	No	13,380	No	12,511 (93.5%)^d^	<0.001	1,196 (9.6%)	<0.001
				Yes	869 (6.5%)^e^		98 (11.3%)	
		Yes	1,531	No	1,343 (87.7%)^d^		169 (12.6%)	
				Yes	188 (12.3%)^e^		38 (20.2%)	

Girls								
	4.5 years old	No	14,947	No	12,643 (84.6%)^d^	<0.001	1,106 (8.7%)	<0.001
				Yes	2,304 (15.4%)^e^		263 (11.4%)	
		Yes	2,062	No	1,054 (51.1%)^d^		80 (7.6%)	
				Yes	1,008 (48.9%)^e^		102 (10.1%)	

	7 years old	No	13,316	No	12,958 (97.3%)^d^	<0.001	1,137 (8.8%)	<0.001
				Yes	358 (2.7%)^e^		51 (14.2%)	
		Yes	1,727	No	1,589 (92.0%)^d^		179 (11.3%)	
				Yes	138 (8.0%)^e^		19 (13.8%)	

	10 years old	No	13,299	No	13,206 (99.3%)^d^	<0.001	1,106 (8.4%)	<0.001
				Yes	93 (0.7%)^e^		17 (18.3%)	
		Yes	1,793	No	1,761 (98.2%)^d^		187 (10.6%)	
				Yes	32 (1.8%)^e^		4 (12.5%)	

	13 years old	No	12,265	No	11,466 (93.3%)^d^	<0.001	611 (5.3%)	<0.001
				Yes	799 (6.5%)^e^		72 (9.0%)	
		Yes	1,596	No	1,406 (88.1%)^d^		92 (6.5%)	
				Yes	190 (11.9%)^e^		23 (12.1%)	

^a^The prevalence is (skipping breakfast at each age (yes/no))/(2.5 years skipping breakfast (yes/no)).

^b^Chi-square test.

^c^The prevalence is (overweight/obese at each age)/(skipping breakfast at each age (yes/no)).

^d,e^|Z| > 1.96 (residual analysis).

Distribution of characteristics according to overweight/obesity status in boys and girls is shown in eTable 1 and eTable 2, respectively. Multivariable adjusted odds ratios of overweight/obesity for skipping breakfast at 2.5 years old are shown in Table 4. There was no condition index greater than 30 and there were not at least two variables with variance decomposition proportions greater than 0.5, indicating no multicollinearity in the models. In the partially adjusted models (models 1 and 2), skipping breakfast at 2.5 years old was not significantly associated with overweight/obesity at 2.5 and 4.5 years old, but was significantly associated with overweight/obesity after 7 years old, in both boys and girls. In the fully adjusted model (model 3), gender difference was observed at 13 years old. Skipping breakfast at 2.5 years old was significantly associated with overweight/obesity at 13 years old in boys (OR 1.38; 95% CI, 1.17–1.62), but not in girls (OR 1.21; 95% CI, 0.98–1.49). Full results (odds ratios of all factors used in the analyses) of model 3 are shown in eTable 3 (boys) and eTable 4 (girls). Significant associations between birth weight of ≥3,500 g and overweight/obesity were observed in all ages and both sexes. Significant positive associations between watching television for more than 3 hours per day and living with a grandparent and overweight/obesity were observed in ≥4.5 years old children in both sexes. In girls, significant negative associations between maternal educational level at junior high school and overweight/obesity were observed.

**Table 4.** Multivariable adjusted odds ratios of overweight/obesity for skipping breakfast at 2.5 years old

		Odds ratio (95% confidence interval) of overweight/obesity				

		2.5 years old	4.5 years old	7 years old	10 years old	13 years old
Boys	Model 1	0.94 (0.79, 1.12)	1.08 (0.90, 1.30)	**1.26 (1.07, 1.48)**	**1.30 (1.12, 1.49)**	**1.40 (1.19, 1.64)**
	Model 2	0.96 (0.81, 1.14)	0.96 (0.79, 1.16)	**1.20 (1.02, 1.41)**	**1.23 (1.07, 1.42)**	**1.43 (1.22, 1.67)**
	Model 3	0.94 (0.79, 1.13)	0.98 (0.81, 1.18)	**1.21 (1.03, 1.43)**	**1.22 (1.06, 1.41)**	**1.38 (1.17, 1.62)**

Girls	Model 1	0.88 (0.75, 1.04)	0.96 (0.81, 1.13)	**1.30 (1.11, 1.53)**	**1.25 (1.06, 1.47)**	**1.24 (1.002, 1.52)**
	Model 2	0.90 (0.77, 1.06)	0.85 (0.72, 1.01)	**1.23 (1.04, 1.44)**	**1.18 (1.003, 1.39)**	**1.27 (1.03, 1.56)**
	Model 3	0.89 (0.76, 1.05)	0.88 (0.74, 1.04)	**1.24 (1.06, 1.47)**	**1.19 (1.01, 1.40)**	1.21 (0.98, 1.49)

Reference group was breakfast eating children at 2.5 years old.

Multivariate logistic regression model.

Model 1: Adjusted for birthweight, breastfeeding exclusiveness, maternal age, paternal age, maternal educational level, and paternal educational level.

Model 2: Adjusted for skipping breakfast (yes/no), hours spent watching television (exclude 13 years old), hours spent playing computer games (exclude 2.5 and 13 years old) and living with a grandparent (yes/no), at each analytical age.

Model 3: Adjusted for factors of Models 1 & 2.

Note: Bold face = statistical significance (*P* < 0.05).

Table 5 shows adjusted ORs for overweight/obesity in relation to skipping breakfast at 2.5 years old, sex and interaction term. In all models, girls were positively associated with overweight/obesity at 2.5 and 4.5 years old, but negatively associated at 10 and 13 years old. Skipping breakfast at 2.5 years old was positively associated with overweight/obesity after 7 years old. The interaction term skipping breakfast and gender was not significant at all ages.

**Table 5.** Multivariable adjusted odds ratios of overweight/obesity for sex and skipping breakfast at 2.5 years old

			Odds ratio (95% confidence interval) of overweight/obesity				

			2.5 years	4.5 years	7 years	10 years	13 years
Model 1	sex	boys (reference)	1	1	1	1	1
		girls	**1.39 (1.29, 1.51)**	**1.64 (1.50, 1.78)**	1.04 (0.96, 1.13)	**0.67 (0.62, 0.73)**	**0.57 (0.52, 0.63)**
	breakfast at 2.5 years old	eating (reference)	1	1	1	1	1
		skipping	0.95 (0.79, 1.13)	1.09 (0.90, 1.31)	**1.25 (1.07, 1.47)**	**1.29 (1.12, 1.49)**	**1.38 (1.17, 1.62)**
	girls and skipping breakfast		0.93 (0.73, 1.18)	0.87 (0.68, 1.12)	1.04 (0.83, 1.31)	0.96 (0.78, 1.20)	0.91 (0.70, 1.18)

Model 2	sex	boys (reference)	1	1	1	1	1
		girls	**1.30 (1.20, 1.41)**	**1.54 (1.42, 1.68)**	1.02 (0.94, 1.11)	**0.68 (0.63, 0.74)**	**0.55 (0.50, 0.61)**
	breakfast at 2.5 years old	eating (reference)	1	1	1	1	1
		skipping	0.96 (0.81, 1.15)	0.97 (0.80, 1.17)	**1.18 (1.01, 1.39)**	**1.22 (1.06, 1.40)**	**1.42 (1.21, 1.66)**
	girls and skipping breakfast		0.93 (0.74, 1.18)	0.87 (0.68, 1.12)	1.05 (0.84, 1.32)	0.99 (0.80, 1.23)	0.91 (0.70, 1.18)

Model 3	sex	boys (reference)	1	1	1	1	1
		girls	**1.40 (1.29, 1.51)**	**1.66 (1.52, 1.80)**	1.06 (0.98, 1.16)	**0.69 (0.64, 0.75)**	**0.57 (0.51, 0.62)**
	breakfast at 2.5 years old	eating (reference)	1	1	1	1	1
		skipping	0.96 (0.80, 1.14)	0.99 (0.82, 1.20)	**1.19 (1.01, 1.41)**	**1.21 (1.05, 1.39)**	**1.35 (1.15, 1.59)**
	girls and skipping breakfast		0.93 (0.73, 1.18)	0.87 (0.68, 1.11)	1.06 (0.84, 1.33)	0.99 (0.80, 1.23)	0.92 (0.71, 1.19)

Reference group was breakfast eating at 2.5 years old.

Multivariate logistic regression analysis.

Model 1: Adjusted for birthweight, breastfeeding exclusiveness, maternal age, paternal age, maternal educational level, and paternal educational level.

Model 2: Adjusted for skipping breakfast (yes/no), hours spent watching television (exclude 13 years old), hours spent playing computer games (exclude 2.5 and 13 years old) and living with a grandparent (yes/no), at each analytical age.

Model 3: Adjusted for factors of Model 1 & 2.

Note: Bold face = statistical significance (*P* < 0.05).

## DISCUSSION

We examined the association between skipping breakfast at 2.5 years old and subsequent overweight/obesity status at 2.5, 4.5, 7, 10, and 13 years old, especially focusing on gender difference. To the best of our knowledge, this is the first study to examine the longitudinal relationship between skipping breakfast and subsequent overweight/obesity from pre-school to 13 years old among an Asian population. Among boys, skipping breakfast at 2.5 years old was significantly associated with overweight/obesity at 7, 10, and 13 years old. Eating habits in the pre-school period may have an independent, long-term impact on obesity status. Among girls, skipping breakfast at 2.5 years old was significantly associated with overweight/obesity at 7 and 10 years old, but not at 13 years old. When both boys and girls were combined and analyzed using the interaction term skipping breakfast and gender, girls were less likely to become obese when they were over 10 years old, and skipping breakfast at 2.5 years old was associated with subsequent overweight/obesity after 7 years old. This finding suggested that the association between childhood dietary habits and subsequent overweight/obesity might largely differ after the junior high school period, especially in girls. A previous Japanese study found that 58% of junior high school girls (26.0% of boys) students wanted to be thinner and 17.3% of female and 5.7% of male students had tried to reduce their weight.^30^ Another previous study found that adolescent girls were more likely to overestimate their own body size, compared with their actual BMI-based weight.^31^ These characteristics of adolescent girls might explain the gender difference seen in junior high school students. Girls might try to reduce their weight, especially after finishing junior high school.

In our study, skipping breakfast at 2.5 years old did not increase overweight/obesity at 2.5 or 4.5 years old. This was consistent with a previous Dutch study: when 4 years old children were followed up after 2 years, skipping breakfast was associated with higher body fat mass, but not associated with increased BMI or body weight.^19^ Consistent with our results, a previous British study of children, followed up after 8 years, found that skipping breakfast at 3 years old was significantly associated with a large increase of BMI in 7 to 11 years old.^17^ On the other hand, an Australian study suggested skipping breakfast to be prospectively associated with overweight in 10–12 years old children after 3 years, but not in 5–6 years old children after 3 years.^9^ The impact of skipping breakfast may be different between children aged up to 3 years old and those aged 5 years or older. Skipping breakfast may have only increased body fat until about 5 years old, but resulted in weight gain (not only body fat) after 7 years old.

Overweight/obesity occurs when energy intake is greater than energy expenditure. Japanese breakfast skipper children (aged 6–11 years old) and adolescents (aged 12–17 years old) had lower intake for vitamins and minerals compared to breakfast consumers.^32^ Furthermore, Japanese breakfast skipper children and adolescents reported higher intake in confectionaries and lower intake in vegetables, fruits, eggs, dairy products.^32^ Some previous studies have indicated that the total daily energy intake of children who skip breakfast was less than that of children who did not skip breakfast, and they did not eat a lot in other meals,^33^^,^^34^ while a previous study found that the total daily energy intake of children who skipped breakfast aged 4–10 years old was similar to that of children who did not skip breakfast.^1^ In a previous study,^10^ children who skipped breakfast were observed to spend a shorter amount of time on physical activity. To elucidate the mechanism between skipping breakfast and subsequent overweight/obesity, further research on total energy intake and physical activity will be necessary. Longitudinal evidence between skipping breakfast and subsequent overweight/obesity has been limited, especially in terms of long-term follow-up. In an Australian study, children aged 9–15 years old were followed up for 20 years. Those who had skipped breakfast as children, were likely to have larger waist circumference, metabolic syndrome and higher BMI.^10^ In a study of Swedish adolescents, those who were 16 years old were followed for 27 years. If they had a lower energy intake, usually including skipping breakfast, they were likely to have central obesity.^12^ Combined with these studies, our findings suggest that skipping breakfast in childhood may have a long-term negative impact on body weight, although careful interpretation and further research are necessary, particularly among adolescent girls older than junior high school age.

There are some limitations in our study. First, dietary assessment including total energy intake, nutrients and food was not collected in the study, so the content of different breakfasts was not known. Moreover, information on physical activity was also not collected in the study. Therefore, we could not determine the energy balance between intake and expenditure, which may indicate a contributing mechanism to overweight/obesity. Second, although the variable of skipping breakfast in each year was adjusted for in the multivariable models, questionnaires for skipping breakfast were different across survey years.^20^ In addition, we need to interpret the results carefully because the status of skipping breakfast has a time-dependent nature: In our results, about 50% of children who skipped breakfast at 2.5 years old ate breakfast at 4.5 years old, and about 90% of subjects who skipped breakfast at 2.5 years old ate breakfast at 7, 10, and 13 years old. These results were consistent with a previous study in which about 75% of breakfast skipper at 4 years old ate breakfast at 6 years old.^19^ Third, the height and weight of the participants were self-reported by their parents. This could potentially lead to misclassification of the results of overweight/obesity.

In conclusion, skipping breakfast in early childhood increased overweight/obesity in later childhood, but there may be gender differences in the association: Although the interaction term skipping breakfast and gender was not significant, a difference in direction of the OR for overweight/obesity across different age groups was observed between boys and girls.

## APPENDIX A. SUPPLEMENTARY DATA

eTable 1. Characteristics according to overweight/obesity status in boys

eTable 2. Characteristics according to overweight/obesity status in girls

eTable 3. Multivariable adjusted odds ratios (95% confidence intervals) of overweight/obesity in boys at 2.5, 4.5, 7, 10, and 13 years old

eTable 4. Multivariable adjusted odds ratios (95% confidence intervals) of overweight/obesity in girls at 2.5, 4.5, 7, 10, and 13 years old
