# Supplementary material for: Skipping Breakfast and Subsequent Overweight/Obesity in Children: A Nationwide Prospective Study of 2.5- to 13-year-old Children in Japan
Source: J Epidemiol. 2021 Jul 5;31(7):417–25. doi: 10.2188/jea.JE20200266 (PMC8187609; doi:10.2188/jea.JE20200266)
Supplement: Supplementary file 1 [file je-31-417-s001.pdf]

eTable 1. Characteristics according to overweight/obesity status in boys

		2.5 years old			4.5 years old			7 years old			10 years old			13 years old		
		Overweight/Obese	Non-overweight/Non-obese	p-value	Overweight/Obese	Non-overweight/Non-obese	p-value	Overweight/Obese	Non-overweight/Non-obese	p-value	Overweight/Obese	Non-overweight/Non-obese	p-value	Overweight/Obese	Non-overweight/Non-obese	p-value
		n=1432	n=16581		n=1161	n=17302		n=1500	n=14723		n=2066	n=14053		n=1501	n=13410	
Birthweight, g [a]	<2500	73 (5.1%)	1297 (7.8%)	<0.001	36 (3.1%)	1355 (7.8%)	<0.001	69 (4.6%)	1146 (7.8%)	<0.001	127 (6.1%)	1082 (7.7%)	<0.001	86 (5.7%)	1002 (7.5%)	<0.001
	2500–2999	387 (27.0%)	5581 (33.7%)		292 (25.2%)	5850 (33.8%)		437 (29.1%)	4991 (33.9%)		611 (29.6%)	4701 (33.5%)		454 (30.2%)	4500 (33.6%)	
	3000–3499	657 (45.9%)	7382 (44.5%)		551 (47.5%)	7676 (44.4%)		661 (44.1%)	6540 (44.4%)		927 (44.9%)	6294 (44.8%)		682 (45.4%)	5998 (44.7%)	
	≥3500	314 (21.9%)	2316 (14.0%)		282 (24.3%)	2414 (14.0%)		333 (22.2%)	2041 (13.9%)		400 (19.4%)	1972 (14.0%)		278 (18.5%)	1908 (14.2%)	
	Missing	1 (0.1%)	5 (0.0%)		0 (0.0%)	7 (0.0%)		0 (0.0%)	5 (0.0%)		1 (0.0%)	4 (0.0%)		1 (0.1%)	2 (0.0%)	
Breastfeeding exclusiveness [b]	Exclusive breastfeeding	288 (20.1%)	3522 (21.2%)	0.169	236 (20.3%)	3746 (21.7%)	0.006	296 (19.7%)	3219 (21.9%)	0.008	410 (19.8%)	3147 (22.4%)	0.005	305 (20.3%)	3023 (22.5%)	0.007
	Mixed feeding	1045 (73.0%)	12097 (73.0%)		833 (71.7%)	12569 (72.6%)		1100 (73.3%)	10726 (72.9%)		1522 (73.7%)	10165 (72.3%)		1092 (72.8%)	9681 (72.2%)	
	Exclusive formula feeding	99 (6.9%)	962 (5.8%)		92 (7.9%)	987 (5.7%)		104 (6.9%)	778 (5.3%)		134 (6.5%)	741 (5.3%)		104 (6.9%)	706 (5.3%)	
Maternal age at birth [a]	≤24	149 (10.4%)	1394 (8.4%)	0.029	109 (9.4%)	1443 (8.3%)	0.619	111 (7.4%)	1136 (7.7%)	0.893	174 (8.4%)	995 (7.1%)	<0.001	124 (8.3%)	933 (7.0%)	0.004
	25–29	454 (31.7%)	5686 (34.3%)		390 (33.6%)	5948 (34.4%)		503 (33.5%)	5028 (34.2%)		629 (30.4%)	4853 (34.5%)		452 (30.1%)	4580 (34.2%)	
	30–34	595 (41.6%)	6743 (40.7%)		467 (40.2%)	7061 (40.8%)		624 (41.6%)	6067 (41.2%)		851 (41.2%)	5859 (41.7%)		635 (42.3%)	5599 (41.8%)	
	≥35	234 (16.3%)	2758 (16.6%)		195 (16.8%)	2850 (16.5%)		262 (17.5%)	2492 (16.9%)		412 (19.9%)	2346 (16.7%)		290 (19.3%)	2298 (17.1%)	
Paternal age at birth [a]	≤24	91 (6.4%)	855 (5.2%)	0.089	59 (5.1%)	895 (5.2%)	0.016	76 (5.1%)	690 (4.7%)	0.002	115 (5.6%)	602 (4.3%)	<0.001	75 (5.0%)	585 (4.4%)	0.001
	25–29	322 (22.5%)	4041 (24.4%)		269 (23.2%)	4221 (24.4%)		339 (22.6%)	3581 (24.3%)		427 (20.7%)	3412 (24.3%)		312 (20.8%)	3152 (23.5%)	
	30–34	545 (38.1%)	6178 (37.3%)		433 (37.3%)	6486 (37.5%)		520 (34.7%)	5619 (38.2%)		720 (34.8%)	5397 (38.4%)		534 (35.6%)	5145 (38.4%)	
	≥35	456 (31.8%)	5363 (32.3%)		379 (32.6%)	5555 (32.1%)		549 (36.6%)	4720 (32.1%)		781 (37.8%)	4539 (32.3%)		566 (37.7%)	4433 (33.1%)	
	Missing	18 (1.3%)	144 (0.9%)		21 (1.8%)	145 (0.8%)		16 (1.1%)	113 (0.8%)		23 (1.1%)	103 (0.7%)		14 (0.9%)	95 (0.7%)	
Maternal educational level [c]	Junior high school	62 (4.3%)	518 (3.1%)	0.085	50 (4.3%)	508 (2.9%)	0.003	50 (3.3%)	376 (2.6%)	0.004	82 (4.0%)	322 (2.3%)	<0.001	62 (4.1%)	294 (2.2%)	<0.001
	High school	547 (38.2%)	6290 (37.9%)		466 (40.1%)	6603 (38.2%)		608 (40.5%)	5462 (37.1%)		879 (42.5%)	5129 (36.5%)		625 (41.6%)	4912 (36.6%)	
	Junior or career college	591 (41.3%)	6966 (42.0%)		467 (40.2%)	7325 (42.3%)		619 (41.3%)	6364 (43.2%)		823 (39.8%)	6189 (44.0%)		590 (39.3%)	5935 (44.3%)	
	University or higher education	187 (13.1%)	2355 (14.2%)		141 (12.1%)	2470 (14.3%)		190 (12.7%)	2247 (15.3%)		241 (11.7%)	2167 (15.4%)		189 (12.6%)	2066 (15.4%)	
	Other/missing	45 (3.1%)	452 (2.7%)		37 (3.2%)	396 (2.3%)		33 (2.2%)	274 (1.9%)		41 (2.0%)	246 (1.8%)		35 (2.3%)	203 (1.5%)	
Paternal educational level [c]	Junior high school	97 (6.8%)	945 (5.7%)	0.287	77 (6.6%)	993 (5.7%)	0.002	93 (6.2%)	764 (5.2%)	<0.001	136 (6.6%)	707 (5.0%)	<0.001	108 (7.2%)	613 (4.6%)	<0.001
	High school	561 (39.2%)	6393 (38.6%)		485 (41.8%)	6657 (38.5%)		636 (42.4%)	5538 (37.6%)		888 (43.0%)	5207 (37.1%)		641 (42.7%)	5004 (37.3%)	
	Junior or career college	212 (14.8%)	2541 (15.3%)		166 (14.3%)	2666 (15.4%)		239 (15.9%)	2261 (15.4%)		339 (16.4%)	2188 (15.6%)		239 (15.9%)	2087 (15.6%)	
	University or higher education	503 (35.1%)	6105 (36.8%)		323 (32.9%)	6450 (37.3%)		491 (32.7%)	5782 (39.3%)		636 (30.8%)	5608 (39.9%)		469 (31.2%)	5419 (40.4%)	
	Other/missing	59 (4.1%)	597 (3.6%)		51 (4.4%)	536 (3.1%)		41 (2.7%)	378 (2.6%)		67 (3.2%)	343 (2.4%)		44 (2.9%)	287 (2.1%)	
Skipping breakfast [d]	No	1280 (89.4%)	14749 (89.0%)	0.615	880 (75.8%)	14064 (81.3%)	<0.001	1438 (95.9%)	14234 (96.7%)	0.098	2043 (98.9%)	13938 (99.2%)	0.174	1365 (90.9%)	12489 (93.1%)	0.002
	Yes	152 (10.6%)	1832 (11.0%)		281 (24.2%)	3238 (18.7%)		62 (4.1%)	489 (3.3%)		23 (1.1%)	115 (0.8%)		136 (9.1%)	921 (6.9%)	
Living with a grandparent [d]	No	1086 (75.8%)	12866 (77.6%)	0.127	839 (72.3%)	13364 (77.2%)	<0.001	1040 (69.3%)	11279 (76.6%)	<0.001	1434 (69.4%)	10968 (78.0%)	<0.001	1061 (70.7%)	10627 (79.2%)	<0.001
	Yes	346 (24.2%)	3715 (22.4%)		322 (27.7%)	3938 (22.8%)		460 (30.7%)	3444 (23.4%)		632 (30.6%)	3085 (22.0%)		440 (29.3%)	2783 (20.8%)	
Watching television, hours per day [d]	<1	166 (11.6%)	1693 (10.2%)	0.293	86 (7.4%)	1919 (11.1%)	<0.001	296 (19.7%)	4154 (28.2%)	<0.001	256 (12.4%)	2878 (20.5%)	<0.001	-	-	
	1–<2	534 (37.3%)	6104 (36.8%)		304 (26.2%)	5142 (29.7%)		688 (45.9%)	6724 (45.7%)		823 (39.8%)	6175 (43.9%)		-	-	
	2–<3	187 (13.1%)	2349 (14.2%)		415 (35.7%)	6153 (35.6%)		373 (24.9%)	2973 (20.2%)		653 (31.6%)	3544 (25.2%)		-	-	
	≥3	510 (35.6%)	6090 (36.7%)		342 (29.5%)	3913 (22.6%)		140 (9.3%)	847 (5.8%)		325 (15.7%)	1406 (10.0%)		-	-	
	Missing	35 (2.4%)	345 (2.1%)		14 (1.2%)	175 (1.0%)		3 (0.2%)	25 (0.2%)		9 (0.4%)	50 (0.4%)		-	-	
Playing computer games, hours per day [d]	<1	-	-		1021 (87.9%)	15557 (89.9%)	0.088	1117 (74.5%)	11632 (79.0%)	<0.001	1239 (60.0%)	9729 (69.2%)	<0.001	-	-	
	1–<2	-	-		103 (8.9%)	1361 (7.9%)		314 (20.9%)	2588 (17.6%)		665 (32.2%)	3511 (25.0%)		-	-	
	≥2	-	-		34 (2.9%)	345 (2.0%)		55 (3.7%)	425 (2.9%)		150 (7.3%)	713 (5.1%)		-	-	
	Missing	-	-		3 (0.3%)	39 (0.2%)		14 (0.9%)	78 (0.5%)		12 (0.6%)	100 (0.7%)		-	-	

chi-square test. [a]: This information collected from birth records. [b]: This information collected from survey at 0.5 years old. [c]: This information collected from survey at 1.5 years old. [d]: This information collected from each analytical survey.

eTable 2. Characteristics according to overweight/obesity status in girls

		2.5years old			4.5 years old			7 years old			10 years old			13 years old		
		Overweight/Obese	Non-overweight/Non-obese	p-value	Overweight/Obese	Non-overweight/Non-obese	p-value	Overweight/Obese	Non-overweight/Non-obese	p-value	Overweight/Obese	Non-overweight/Non-obese	p-value	Overweight/Obese	Non-overweight/Non-obese	p-value
		n=1667	n=14969		n=1551	n=15458		n=1386	n=13657		n=1314	n=13778		n=798	n=13063	
Birthweight, g [a]	<2500	90 (5.4%)	1493 (10.0%)	<0.001	85 (5.5%)	1493 (9.7%)	<0.001	82 (5.9%)	1284 (9.4%)	<0.001	81 (6.2%)	1285 (9.3%)	<0.001	49 (6.1%)	1202 (9.2%)	<0.001
	2500–2999	524 (31.4%)	6214 (41.5%)		509 (32.8%)	6389 (41.3%)		455 (32.8%)	5605 (41.0%)		465 (35.4%)	5630 (40.9%)		275 (34.5%)	5319 (40.7%)	
	3000–3499	773 (46.4%)	5935 (39.6%)		693 (44.7%)	6205 (40.1%)		628 (45.3%)	5538 (40.6%)		577 (43.9%)	5630 (40.9%)		364 (45.6%)	5350 (41.0%)	
	≥3500	280 (16.8%)	1324 (8.8%)		264 (17.0%)	1369 (8.9%)		221 (15.9%)	1230 (9.0%)		191 (14.5%)	1231 (8.9%)		110 (13.8%)	1190 (9.1%)	
	Missing	0 (0.0%)	3 (0.0%)		0 (0.0%)	2 (0.0%)		0 (0.0%)	0 (0.0%)		0 (0.0%)	2 (0.0%)		0 (0.0%)	2 (0.0%)	
Breastfeeding exclusiveness [b]	Exclusive breastfeeding	345 (20.7%)	3323 (22.2%)	0.274	349 (22.5%)	3456 (22.4%)	0.343	310 (22.4%)	3155 (23.1%)	0.032	276 (21.0%)	3201 (23.2%)	0.003	166 (20.8%)	3021 (23.1%)	0.001
	Mixed feeding	1221 (73.2%)	10824 (72.3%)		1102 (71.1%)	11141 (72.1%)		978 (70.6%)	9766 (71.5%)		940 (71.5%)	9833 (71.4%)		568 (71.2%)	9367 (71.7%)	
	Exclusive formula feeding	101 (6.1%)	822 (5.5%)		100 (6.4%)	861 (5.6%)		98 (7.1%)	736 (5.4%)		98 (7.5%)	744 (5.4%)		64 (8.0%)	675 (5.2%)	
Maternal age at birth [a]	≤24	153 (9.2%)	1273 (8.5%)	0.818	123 (7.9%)	1278 (8.3%)	0.036	110 (7.9%)	1001 (7.3%)	0.010	94 (7.2%)	1034 (7.5%)	0.001	73 (9.1%)	901 (6.9%)	<0.001
	25–29	567 (34.0%)	5104 (34.1%)		503 (32.4%)	5330 (34.5%)		420 (30.3%)	4708 (34.5%)		401 (30.5%)	4685 (34.0%)		244 (30.6%)	4375 (33.5%)	
	30–34	667 (40.0%)	6079 (40.6%)		627 (40.4%)	6311 (40.8%)		592 (42.7%)	5659 (41.4%)		544 (41.4%)	5760 (41.8%)		298 (37.3%)	5563 (42.6%)	
	≥35	280 (16.8%)	2513 (16.8%)		298 (19.2%)	2539 (16.4%)		264 (19.0%)	2289 (16.8%)		275 (20.9%)	2299 (16.7%)		183 (22.9%)	2224 (17.0%)	
Paternal age at birth [a]	≤24	95 (5.7%)	762 (5.1%)	0.218	69 (4.4%)	758 (4.9%)	0.247	63 (4.5%)	595 (4.4%)	0.013	52 (4.0%)	632 (4.6%)	<0.001	41 (5.1%)	541 (4.1%)	0.003
	25–29	393 (23.6%)	3723 (24.9%)		373 (24.0%)	3834 (24.8%)		302 (21.8%)	3394 (24.9%)		281 (21.4%)	3383 (24.6%)		157 (19.7%)	3133 (24.0%)	
	30–34	598 (35.9%)	5603 (37.4%)		563 (36.3%)	5842 (37.8%)		508 (36.7%)	5203 (38.1%)		470 (35.8%)	5262 (38.2%)		288 (36.1%)	5027 (38.5%)	
	≥35	564 (33.8%)	4715 (31.5%)		532 (34.3%)	4868 (31.5%)		500 (36.1%)	4351 (31.9%)		498 (37.8%)	4384 (31.8%)		304 (38.1%)	4258 (32.6%)	
	Missing	17 (1.0%)	166 (1.1%)		14 (0.9%)	156 (1.0%)		13 (0.9%)	114 (0.8%)		13 (1.0%)	117 (0.8%)		8 (1.0%)	104 (0.8%)	
Maternal educational level [c]	Junior high school	75 (4.5%)	483 (3.2%)	<0.001	66 (4.3%)	457 (3.0%)	0.001	63 (4.5%)	348 (2.5%)	<0.001	66 (5.0%)	322 (2.3%)	<0.001	44 (5.5%)	302 (2.3%)	<0.001
	High school	641 (38.5%)	5741 (38.4%)		640 (41.3%)	5950 (38.5%)		605 (43.7%)	5081 (37.2%)		617 (47.0%)	5106 (37.1%)		369 (46.2%)	4797 (36.7%)	
	Junior or career college	621 (37.3%)	6120 (40.9%)		610 (39.3%)	6384 (41.3%)		525 (37.9%)	5788 (42.4%)		486 (37.0%)	5879 (42.7%)		282 (35.3%)	5655 (43.3%)	
	University or higher education	264 (15.8%)	2242 (15.0%)		197 (12.7%)	2337 (15.1%)		161 (11.6%)	2196 (16.1%)		126 (9.6%)	2235 (16.2%)		88 (11.0%)	2127 (16.3%)	
	Other/missing	66 (4.0%)	383 (2.6%)		38 (2.5%)	330 (2.1%)		32 (2.3%)	244 (1.8%)		19 (1.4%)	236 (1.7%)		15 (1.9%)	182 (1.4%)	
Paternal educational level [c]	Junior high school	126 (7.6%)	818 (5.5%)	0.001	96 (6.2%)	837 (5.4%)	<0.001	80 (5.8%)	678 (5.0%)	<0.001	96 (7.3%)	679 (4.9%)	<0.001	56 (7.0%)	598 (4.6%)	<0.001
	High school	633 (38.0%)	5753 (38.4%)		659 (42.5%)	5911 (38.2%)		572 (41.3%)	5185 (38.0%)		600 (45.7%)	5141 (37.3%)		367 (46.0%)	4915 (37.6%)	
	Junior or career college	261 (15.7%)	2292 (15.3%)		223 (14.4%)	2407 (15.6%)		220 (15.9%)	2111 (15.5%)		201 (15.3%)	2127 (15.4%)		119 (14.9%)	2016 (15.4%)	
	University or higher education	572 (34.3%)	5562 (37.2%)		512 (33.0%)	5831 (37.7%)		463 (33.4%)	5334 (39.1%)		380 (28.9%)	5489 (39.8%)		232 (29.1%)	5253 (40.2%)	
	Other/missing	75 (4.5%)	544 (3.6%)		61 (3.9%)	472 (3.1%)		51 (3.7%)	349 (2.6%)		37 (2.8%)	342 (2.5%)		24 (3.0%)	281 (2.1%)	
Skipping breakfast [d]	No	1480 (88.8%)	13119 (87.6%)	0.178	1186 (76.5%)	12511 (80.9%)	<0.001	1316 (94.9%)	13231 (96.9%)	<0.001	1293 (98.4%)	13674 (99.2%)	0.001	703 (88.1%)	12169 (93.2%)	<0.001
	Yes	187 (11.2%)	1850 (12.4%)		365 (23.5%)	2947 (19.1%)		70 (5.1%)	426 (3.1%)		21 (1.6%)	104 (0.8%)		95 (11.9%)	894 (6.8%)	
Living with a grandparent [d]	No	1221 (73.2%)	11686 (78.1%)	<0.001	1123 (72.4%)	11958 (77.4%)	<0.001	976 (70.4%)	10522 (77.0%)	<0.001	912 (69.4%)	10802 (78.4%)	<0.001	573 (71.8%)	10339 (79.1%)	<0.001
	Yes	446 (26.8%)	3283 (21.9%)		428 (27.6%)	3500 (22.6%)		410 (29.6%)	3135 (23.0%)		402 (30.6%)	2976 (21.6%)		225 (28.2%)	2724 (20.9%)	
Watching television, hours per day [d]	<1	203 (12.2%)	1489 (9.9%)	<0.001	166 (10.7%)	1973 (12.8%)	<0.001	321 (23.2%)	4129 (30.2%)	<0.001	156 (11.9%)	2897 (21.0%)	<0.001	-	-	
	1–<2	668 (40.1%)	5472 (36.6%)		434 (28.0%)	4728 (30.6%)		573 (41.3%)	6048 (44.3%)		453 (34.5%)	5801 (42.1%)		-	-	
	2–<3	202 (12.1%)	2138 (14.3%)		519 (33.5%)	5296 (34.3%)		354 (25.5%)	2671 (19.6%)		434 (33.0%)	3566 (25.9%)		-	-	
	≥3	556 (33.4%)	5591 (37.4%)		411 (26.5%)	3310 (21.4%)		134 (9.7%)	789 (5.8%)		266 (20.2%)	1476 (10.7%)		-	-	
	Missing	38 (2.3%)	279 (1.9%)		21 (1.4%)	151 (1.0%)		4 (0.3%)	20 (0.1%)		5 (0.4%)	38 (0.3%)		-	-	
Playing computer games, hours per day [d]	<1	-	-		1481 (95.5%)	14865 (96.2%)	0.172	1223 (88.2%)	12593 (92.2%)	<0.001	1082 (82.3%)	12173 (88.4%)	<0.001	-	-	
	1–<2	-	-		57 (3.7%)	471 (3.0%)		137 (9.9%)	885 (6.5%)		193 (14.7%)	1385 (10.1%)		-	-	
	≥2	-	-		13 (0.8%)	99 (0.6%)		9 (0.6%)	84 (0.6%)		30 (2.3%)	149 (1.1%)		-	-	
	Missing	-	-		0 (0.0%)	23 (0.1%)		17 (1.2%)	95 (0.7%)		9 (0.7%)	71 (0.5%)		-	-	

chi-square test. [a]: This information collected from birth records. [b]: This information collected from survey at 0.5 years old. [c]: This information collected from survey at 1.5 years old. [d]: This information collected from each analytical survey.

eTable 3. Multivariable adjusted odds ratios (95% confidence intervals) of overweight/obesity in boys at 2.5, 4.5, 7, 10, and 13 years old

		2.5years old	4.5 years old	7 years old	10 years old	13 years old
Skipping breakfast at 2.5 years old [a]	No	1	1	1	1	1
	Yes	0.94 (0.79, 1.13)	0.98 (0.81, 1.18)	**1.21 (1.03, 1.43)**	**1.22 (1.06, 1.41)**	**1.38 (1.17, 1.62)**
Birthweight, g [b]	<2500	0.80 (0.62, 1.03)	**0.52 (0.36, 0.74)**	**0.67 (0.51, 0.87)**	0.86 (0.70, 1.05)	0.79 (0.62, 1.01)
	2500–2999	1	1	1	1	1
	3000–3499	**1.29 (1.13, 1.47)**	**1.45 (1.25, 1.68)**	**1.17 (1.03, 1.33)**	**1.13 (1.01, 1.26)**	1.13 (0.998, 1.28)
	≥3500	**1.97 (1.68, 2.30)**	**2.36 (1.99, 2.80)**	**1.86 (1.60, 2.17)**	**1.52 (1.33, 1.75)**	**1.42 (1.21, 1.66)**
	Missing	2.82 (0.33, 24.3)	NA	NA	1.43 (0.15, 13.4)	3.88 (0.34, 43.9)
Breastfeeding exclusiveness [c]	Exclusive breastfeeding	1	1	1	1	1
	Mixed feeding	1.08 (0.94, 1.24)	1.04 (0.90, 1.21)	1.10 (0.96, 1.26)	1.09 (0.96, 1.22)	1.08 (0.94, 1.24)
	Exclusive formula feeding	1.27 (0.998, 1.63)	**1.35 (1.04, 1.75)**	**1.31 (1.03, 1.67)**	1.13 (0.91, 1.40)	1.22 (0.96, 1.56)
Maternal age at birth [b]	≤24	1	1	1	1	1
	25–29	0.82 (0.65, 1.03)	0.94 (0.73, 1.23)	1.23 (0.95, 1.59)	0.97 (0.78, 1.21)	0.88 (0.69, 1.14)
	30–34	0.88 (0.68, 1.13)	0.92 (0.70, 1.22)	1.22 (0.93, 1.61)	1.05 (0.84, 1.33)	0.99 (0.76, 1.30)
	≥35	0.85 (0.64, 1.13)	0.93 (0.68, 1.28)	1.08 (0.80, 1.46)	1.14 (0.88, 1.48)	1.01 (0.75, 1.36)
Paternal age at birth [b]	≤24	1	1	1	1	1
	25–29	0.88 (0.66, 1.17)	1.15 (0.83, 1.61)	0.87 (0.64, 1.19)	0.78 (0.60, 1.01)	0.97 (0.71, 1.32)
	30–34	0.97 (0.72, 1.31)	1.29 (0.91, 1.82)	0.89 (0.65, 1.23)	0.86 (0.66, 1.13)	1.06 (0.77, 1.45)
	≥35	0.92 (0.67, 1.26)	1.30 (0.90, 1.86)	1.16 (0.84, 1.61)	1.07 (0.81, 1.41)	1.27 (0.91, 1.76)
	Missing	1.23 (0.69, 2.21)	**2.27 (1.26, 4.08)**	1.35 (0.73, 2.51)	1.02 (0.59, 1.76)	1.18 (0.61, 2.27)
Maternal educational level [d]	Junior high school	1.26 (0.95, 1.69)	1.19 (0.86, 1.64)	1.13 (0.82, 1.55)	**1.36 (1.05, 1.77)**	**1.44 (1.07, 1.95)**
	High school	1	1	1	1	1
	Junior or career college	1.02 (0.90, 1.16)	1.03 (0.90, 1.19)	1.00 (0.89, 1.14)	0.91 (0.82, 1.02)	0.89 (0.78, 1.01)
	University or higher education	0.96 (0.79, 1.16)	1.02 (0.82, 1.27)	1.02 (0.84, 1.23)	0.93 (0.79, 1.10)	0.93 (0.77, 1.13)
	Others/missing	1.17 (0.67, 2.03)	1.30 (0.72, 2.33)	1.51 (0.82, 2.77)	0.77 (0.46, 1.29)	1.46 (0.81, 2.65)
Paternal educational level [d]	Junior high school	1.11 (0.88, 1.40)	1.04 (0.80, 1.34)	1.07 (0.84, 1.35)	1.07 (0.88, 1.31)	**1.33 (1.06, 1.67)**
	High school	1	1	1	1	1
	Junior or career college	0.96 (0.81, 1.13)	0.87 (0.72, 1.04)	0.96 (0.82, 1.13)	0.99 (0.86, 1.13)	0.95 (0.81, 1.12)
	University or higher education	0.97 (0.84, 1.12)	0.88 (0.75, 1.03)	**0.80 (0.70, 0.92)**	**0.77 (0.68, 0.87)**	**0.72 (0.63, 0.83)**
	Others/missing	0.96 (0.58, 1.57)	1.04 (0.62, 1.74)	0.72 (0.42, 1.25)	1.35 (0.88, 2.05)	0.89 (0.52, 1.52)
Skipping breakfast at each analytical age [e]	No	1	1	1	1	1
	Yes	0.94 (0.79, 1.13)	**1.27 (1.10, 1.48)**	1.03 (0.78, 1.36)	0.99 (0.63, 1.57)	**1.21 (1.001, 1.47)**
Living with a grandparent [e]	No	1	1	1	1	1
	Yes	1.07 (0.94, 1.21)	**1.25 (1.09, 1.44)**	**1.37 (1.22, 1.55)**	**1.46 (1.31, 1.62)**	**1.50 (1.33, 1.70)**
Watching television, hours per day [e]	<1	1	1	1	1	NA
	1–<2	0.90 (0.75, 1.08)	1.27 (0.995, 1.63)	**1.36 (1.18, 1.57)**	**1.36 (1.17, 1.57)**	NA
	2–<3	0.81 (0.65, 1.01)	**1.43 (1.12, 1.82)**	**1.62 (1.38, 1.91)**	**1.74 (1.49, 2.04)**	NA
	≥3	0.86 (0.71, 1.03)	**1.80 (1.40, 2.31)**	**2.07 (1.65, 2.58)**	**2.08 (1.73, 2.51)**	NA
	Missing	1.03 (0.70, 1.52)	1.52 (0.84, 2.77)	1.23 (0.35, 4.31)	2.29 (0.96, 5.45)	NA
Playing computer games, hours per day [e]	<1	NA	1	1	1	NA
	1–<2	NA	1.00 (0.81, 1.24)	1.11 (0.97, 1.27)	**1.25 (1.12, 1.39)**	NA
	≥2	NA	1.23 (0.85, 1.78)	1.04 (0.77, 1.40)	**1.22 (1.002, 1.48)**	NA
	Missing	NA	1.08 (0.33, 3.54)	**1.84 (1.01, 3.36)**	0.66 (0.32, 1.36)	NA

Multivariate logistic regression model.

Adjusted for skipping breakfast at 2.5 years old (yes/no), birthweight, breastfeeding exclusiveness, maternal age at birth, paternal age at birth, maternal educational level, paternal educational level, skipping breakfast (yes/no), hours spent watching television at each age (excluding 13 years old), hours spent playing computer games (exclude 2.5 and 13 years old) and living with a grandparent (yes/no), at each analytical age.

[a]: This information collected from the survey at 2.5 years old. [b]: This information collected from birth records. [c]: This information collected from the survey at 0.5 years old. [d]: This information collected from the survey at 1.5 years old. [e]: This information collected from each survey.

Note: Bold face = statistical significance ($p<0.05$). NA = not applicable.

eTable 4. Multivariable adjusted odds ratios (95% confidence intervals) of overweight/obesity in girls at 2.5, 4.5, 7, 10, and 13 years old

		2.5years old	4.5 years old	7 years old	10 years old	13 years old
Skipping breakfast at 2.5 years old [a]	No	1	1	1	1	1
	Yes	0.89 (0.76, 1.05)	0.88 (0.74, 1.04)	**1.24 (1.06, 1.47)**	**1.19 (1.01, 1.40)**	1.21 (0.98, 1.49)
Birthweight, g [b]	<2500	**0.69 (0.55, 0.87)**	**0.71 (0.56, 0.89)**	**0.77 (0.60, 0.98)**	**0.74 (0.58, 0.94)**	0.75 (0.55, 1.02)
	2500–2999	1	1	1	1	1
	3000–3499	**1.55 (1.38, 1.74)**	**1.40 (1.24, 1.58)**	**1.40 (1.23, 1.59)**	**1.24 (1.09, 1.42)**	**1.31 (1.11, 1.54)**
	≥3500	**2.49 (2.13, 2.92)**	**2.39 (2.03, 2.81)**	**2.14 (1.80, 2.55)**	**1.76 (1.46, 2.11)**	**1.72 (1.36, 2.16)**
	Missing	NA	NA	NA	NA	NA
Breastfeeding exclusiveness [c]	Exclusive breastfeeding	1	1	1	1	1
	Mixed feeding	**1.14 (1.001, 1.29)**	0.97 (0.86, 1.11)	0.99 (0.87, 1.14)	1.04 (0.90, 1.20)	1.05 (0.88, 1.26)
	Exclusive formula feeding	1.27 (0.996, 1.61)	1.10 (0.87, 1.40)	1.24 (0.97, 1.59)	1.26 (0.98, 1.62)	**1.45 (1.06, 1.97)**
Maternal age at birth [b]	≤24	1	1	1	1	1
	25–29	1.00 (0.79, 1.25)	1.02 (0.79, 1.30)	0.95 (0.73, 1.23)	1.09 (0.82, 1.43)	0.82 (0.59, 1.13)
	30–34	0.92 (0.72, 1.18)	1.08 (0.83, 1.40)	1.08 (0.82, 1.43)	1.16 (0.87, 1.56)	0.73 (0.52, 1.03)
	≥35	0.87 (0.66, 1.14)	1.25 (0.94, 1.67)	1.11 (0.82, 1.51)	1.37 (0.997, 1.89)	1.04 (0.71, 1.51)
Paternal age at birth [b]	≤24	1	1	1	1	1
	25–29	0.94 (0.71, 1.24)	1.21 (0.89, 1.66)	1.01 (0.72, 1.41)	1.22 (0.86, 1.74)	1.00 (0.66, 1.51)
	30–34	1.01 (0.76, 1.35)	1.21 (0.88, 1.67)	1.08 (0.77, 1.53)	1.39 (0.96, 2.00)	1.34 (0.87, 2.05)
	≥35	1.15 (0.85, 1.55)	1.26 (0.90, 1.76)	1.21 (0.85, 1.73)	**1.59 (1.09, 2.32)**	1.46 (0.94, 2.28)
	Missing	0.92 (0.51, 1.64)	0.75 (0.39, 1.44)	0.79 (0.39, 1.57)	0.95 (0.47, 1.92)	1.06 (0.45, 2.48)
Maternal educational level [d]	Junior high school	**1.31 (1.003, 1.71)**	1.32 (0.996, 1.75)	**1.46 (1.09, 1.96)**	**1.62 (1.21, 2.17)**	**1.62 (1.14, 2.29)**
	High school	1	1	1	1	1
	Junior or career college	0.95 (0.84, 1.07)	0.98 (0.87, 1.11)	**0.84 (0.74, 0.96)**	**0.81 (0.71, 0.92)**	**0.74 (0.63, 0.87)**
	University or higher education	1.16 (0.97, 1.37)	0.94 (0.78, 1.13)	**0.74 (0.60, 0.90)**	**0.67 (0.54, 0.83)**	**0.70 (0.54, 0.90)**
	Other/missing	**2.29 (1.30, 4.02)**	0.70 (0.40, 1.22)	0.78 (0.43, 1.43)	0.52 (0.26, 1.03)	0.92 (0.41, 2.07)
Paternal educational level [d]	Junior high school	**1.34 (1.08, 1.65)**	1.01 (0.80, 1.27)	1.00 (0.77, 1.29)	1.11 (0.87, 1.41)	1.09 (0.80, 1.48)
	High school	1	1	1	1	1
	Junior or career college	1.06 (0.90, 1.23)	**0.85 (0.72, 0.997)**	1.03 (0.87, 1.22)	0.89 (0.75, 1.05)	0.88 (0.71, 1.10)
	University or higher education	0.93 (0.82, 1.07)	**0.85 (0.74, 0.97)**	0.95 (0.82, 1.10)	**0.75 (0.65, 0.87)**	**0.69 (0.58, 0.84)**
	Other/missing	0.65 (0.38, 1.12)	1.54 (0.97, 2.45)	1.52 (0.92, 2.53)	1.35 (0.79, 2.30)	1.07 (0.55, 2.10)
Skipping breakfast at each analytical age [e]	No	1	1	1	1	1
	Yes	0.89 (0.76, 1.05)	**1.25 (1.09, 1.43)**	**1.34 (1.03, 1.75)**	1.40 (0.85, 2.28)	**1.63 (1.30, 2.05)**
Living with a grandparent [e]	No	1	1	1	1	1
	Yes	**1.26 (1.12, 1.42)**	**1.26 (1.11, 1.42)**	**1.35 (1.19, 1.53)**	**1.51 (1.33, 1.71)**	**1.40 (1.19, 1.65)**
Watching television, hours per day [e]	<1	1	1	1	1	NA
	1–<2	0.89 (0.75, 1.05)	1.06 (0.88, 1.29)	1.13 (0.98, 1.30)	**1.28 (1.06, 1.55)**	NA
	2–<3	**0.69 (0.56, 0.85)**	1.11 (0.92, 1.34)	**1.49 (1.27, 1.76)**	**1.85 (1.52, 2.25)**	NA
	≥3	**0.74 (0.62, 0.87)**	**1.40 (1.15, 1.70)**	**1.83 (1.46, 2.29)**	**2.62 (2.11, 3.25)**	NA
	Missing	0.92 (0.63, 1.34)	1.60 (0.97, 2.65)	1.70 (0.55, 5.28)	2.12 (0.72, 6.21)	NA
Playing computer games, hours per day [e]	<1	NA	1	1	1	NA
	1–<2	NA	1.08 (0.81, 1.43)	**1.31 (1.07, 1.59)**	1.18 (0.995, 1.39)	NA
	≥2	NA	1.08 (0.59, 1.97)	0.73 (0.36, 1.47)	1.39 (0.92, 2.10)	NA
	Missing	NA	NA	1.59 (0.93, 2.73)	1.05 (0.47, 2.33)	NA

Multivariate logistic regression model.

Adjusted for skipping breakfast at 2.5 years old (yes/no), birthweight, breastfeeding exclusiveness, maternal age at birth, paternal age at birth, maternal educational level, paternal educational level, skipping breakfast (yes/no), hours spent watching television at each age (excluding 13 years old), hours spent playing computer games (exclude 2.5 and 13 years old) and living with a grandparent (yes/no), at each analytical age.

[a]: This information collected from the survey at 2.5 years old. [b]: This information collected from birth records. [c]: This information collected from the survey at 0.5 years old. [d]: This information collected from the survey at 1.5 years old. [e]: This information collected from each survey.

Note: Bold face = statistical significance (p<0.05). NA = not applicable.
